# Preferences for Renal Cell Carcinoma Pharmacological Treatment: A Discrete Choice Experiment in Patients and Oncologists

**DOI:** 10.3389/fonc.2021.773366

**Published:** 2022-01-07

**Authors:** Ovidio Fernández, Martín Lázaro-Quintela, Guillermo Crespo, Diego Soto de Prado, Álvaro Pinto, Laura Basterretxea, Alfonso Gómez de Liaño, Olatz Etxaniz, Sara Blasco, Clara Gabás-Rivera, Susana Aceituno, Virginia Palomar, Carlos Polanco-Sánchez

**Affiliations:** ^1^ Department of Oncology, Complejo Hospitalario Universitario de Orense, Orense, Spain; ^2^ Department of Oncology, Hospital Álvaro Cunqueiro, Complejo Hospitalario Universitario de Vigo, Pontevedra, Spain; ^3^ Translational Oncology Research Group (ONCO-INVES), Galicia Sur Health Research Institute (IIS Galicia Sur), SERGAS-UVIGO, Pontevedra, Spain; ^4^ Department of Oncology, Complejo Asistencial Universitario de Burgos, Burgos, Spain; ^5^ Department of Oncology, Hospital Clínico Universitario de Valladolid, Valladolid, Spain; ^6^ Department of Oncology, Hospital Universitario La Paz, Madrid, Spain; ^7^ Department of Oncology, Donostialdea ESI/OSI Donostialdea, Donostia, Unibertsitate Ospitalea/Hospital Universitario Donostia, Donostia, Spain; ^8^ Department of Oncology, Complejo Hospitalario Universitario Insular Materno-Infantil, Las Palmas de Gran Canaria, Spain; ^9^ Department of Oncology, Institut Català d’Oncologia, Badalona, Spain; ^10^ Department of Oncology, Hospital de Sagunto, Valencia, Spain; ^11^ Outcomes10, Castellón, Spain; ^12^ Bristol Myers Squibb, Madrid, Spain

**Keywords:** renal cell carcinoma (RCC), kidney cancer, advanced cancer, preferences, discrete-choice experiment (DCE)

## Abstract

**Introduction:**

The purpose of this investigation was to explore patients’ and oncologists’ preferences for the characteristics of a pharmacological regimen for patients with advanced renal cell carcinoma (aRCC).

**Material and Methods:**

Cross-sectional observational study based on a discrete choice experiment (DCE) conducted in Spain. A literature review, a focus group with oncologists and interviews with patients informed the DCE design. Five attributes were included: progression survival gain, risk of serious adverse events (SAEs), health-related quality of life (HRQoL), administration mode, and treatment cost. Preferences were analyzed using a mixed-logit model to estimate relative importance (RI) of attributes (importance of an attribute in relation to all others), which was compared between aRCC patients and oncologists treating aRCC. Willingness to pay (WTP, payer: health system) for a benefit in survival or in risk reduction and maximum acceptable risk (MAR) in SAEs for improving survival were estimated from the DCE. Subgroup analyses were performed to identify factors that influence preference.

**Results:**

A total of 105 patients with aRCC (77.1% male, mean age 65.9 years [SD: 10.4], mean time since RCC diagnosis 6.3 years [SD: 6.1]) and 67 oncologists (52.2% male, mean age 41.9 years [SD: 8.4], mean duration of experience in RCC 10.2 years [SD: 7.5]) participated in the study. The most important attribute for patients and oncologists was survival gain (RI: 43.6% vs. 54.7% respectively, p<0.05), followed by HRQoL (RI: 35.5% vs. 18.0%, respectively, p<0.05). MAR for SAEs was higher among oncologists than patients, while WTP (for the health system) was higher for patients. Differences in preferences were found according to time since diagnosis and education level (patients) or length of professional experience (oncologists).

**Conclusion:**

Patients’ and oncologists’ preferences for aRCC treatment are determined mainly by the efficacy (survival gain) but also by the HRQoL provided. The results of the study can help to inform decision-making in the selection of appropriate aRCC treatment.

## Introduction

Renal cell carcinoma (RCC) is among the most common cancers (1), representing 2.3% of all adult malignancies worldwide (2). RCCs arise from a variety of specialized cells located along the length of the nephron, giving rise to the diversity of histologic RCC types (3). The most common type of RCC is clear-cell RCC, that accounts for up to 75% of RCCs (4).

About 65% of patients with RCC have localized tumors, which are generally treated with surgery and may be cured by total or partial nephrectomy (1, 4). However, the remaining ~35% of patients who present with advanced RCC (aRCC) (which can be partially resected or destructed by surgery) or patients who relapse after local therapy, typically require systemic treatment (1, 5, 6). Nonetheless, aRCC has usually been refractory to conventional cytotoxic chemotherapy (7). Historically, cytokines, known for their limited activity and poor tolerability (8), were the only option available for aRCC. Notably, the management of RCC has been revolutionized in recent years, with the availability of drugs that improve survival for patients with advanced disease dramatically broadening (9, 10).

Compared to cytokines, targeted anti-angiogenic agents have demonstrated superior antitumor activity (11, 12). In addition, aRCC patients treated with sequences of targeted therapies have achieved extended survival (13). Although these treatments have demonstrated a clear progression-free survival benefit, only a small proportion of patients achieve long-term survival, highlighting the need for other therapeutic options with novel mechanisms of action that could potentially result in improved survival (14, 15). The introduction of immune checkpoint inhibitors that alter the interaction between immune cells and antigen-presenting cells, including tumor cells, has changed the treatment landscape for refractory solid tumors (10, 16), and has demonstrated improved overall survival in advanced melanoma (17), non-small-cell lung cancer (18, 19), and aRCC (20). Furthermore, tyrosine kinase inhibitor/immune checkpoint inhibitor combinations appear to be an important treatment option for patients with newly diagnosed aRCC, having shown improved response and overall survival in the first-line setting (21).

Each of the treatments is associated with different clinical outcomes, administration modes, treatment-related adverse events, and costs (22), so decisions about therapy involve tradeoffs between the possible benefits and harms (9). The European Medicines Agency has indicated that in order to improve the management of treatment toxicities, it is important to better understand the extent to which patients are willing to tolerate AEs and, therefore, the relative importance patients give them (23). Additionally, aspects of patient comfort and preference are gaining greater attention when evaluating the best treatment concept for a patient in oncologic treatment (24). In this regard, it is unclear how the specific characteristics of recently approved immunotherapies such as mode and frequency of administration will affect patients and professionals’ preferences when choosing a treatment.

Most cancer patients favor joint decision-making with their doctor, rather than either party make a unilateral decision (25). Shared decision-making is an increasingly used model in medicine, which requires clinicians to understand their patients’ as well as their own preferences (26). For this purpose, there is a need to assess patients’ preferences (27) and enhance patient participation in the decision-making process (28, 29). A discrete choice experiment (DCE) is the stated preference survey format most commonly used to measure preferences for different healthcare interventions, offering a mechanism to facilitate shared decision-making between doctor and patient (30).

Limited evidence exists on which attributes of treatment regimens for aRCC are most important to patients and the extent to which the physician’s judgment matches the patient’s preferences (31–33). Thus, a better understanding of aRCC patients and oncologists’ treatment preferences could help to reduce the knowledge gap between them and consequently improve aRCC management and shared decision-making.

## Materials and Methods

### Study Design and Participants

This was an observational, multicenter, cross-sectional study conducted in Spain among patients with aRCC and oncologists.

Participating patients were recruited in nine Spanish hospitals by their physicians between August 2019 and July 2020. They were aged 18 or older; had been diagnosed with RCC at least two months prior to study inclusion; had aRCC at the time of the study; were receiving or had previously received pharmacological treatment for aRCC (the patient may have received different treatments); and were able to carry out the study tasks.

Oncologists practicing at Spanish public hospitals with more than two years’ experience in RCC management were identified by the sponsor and invited by e-mail to participate. The study questionnaire was available from July to August 2019.

The study was approved by the Ethics Committee of the *Hospital General Universitario Gregorio Marañon (Madrid, Spain)*. Patients provided their written informed consent to participate in the study and oncologists voluntarily accepted to participate. No economic compensation was offered to participants.

The eligible patient population was estimated based on the population over 20 years of age in Spain in 2017 (37,315,882) (34), the prevalence of kidney cancer (0.011%) (35), and the proportion of patients with RCC (80%) (36). Thus, the eligible population for the study was 3,194 RCC patients. The oncologist population was established according to the number of oncologists treating RCC in Spain, based on the scientific committee’s estimate that about 1-2 oncologists per hospital treat RCC (including medium-large hospitals, i.e., >200 beds). Thus, the eligible population was 162 oncologists in Spain (37).

The minimum sample size necessary for the DCE was based on the estimation of proportions, coinciding with an approach proposed by Orme (38). The maximum variation criterion was applied (p=q=0.5), with a 95% confidence interval (Z_α_=1.96) and a 10% accuracy error. Based on this, a minimum necessary sample of 93 aRCC patients and 61 oncologists was estimated.

A steering committee consisting of two oncologists, experts in the management of RCC, led the study and provided scientific advice during its development.

### Discrete Choice Experiment

DCE is a technique for eliciting individuals’ preferences in an indirect way (39), based on the premise that medical interventions can be described as combinations of different features or attributes. DCE involves asking respondents to choose between competing scenarios, each comparing two hypothetical treatment options with a series of defined attributes represented at various levels (e.g., an attribute of ‘route of administration’ at the level of ‘oral’). Using the choice data, the value that individuals attach to their constituent parts is estimated *via* probabilistic choice models, such as logit and probit models (39). DCE was applied according to the International Society for Pharmacoeconomics and Outcomes Research (ISPOR) Good Research Practices for Conjoint Analysis (30).

#### Attributes and Levels Selection

Attributes and their associated levels were initially identified by literature review. Key terms related to the disease (RCC), its treatment or treatment-related decision-making, and preferences studies were used to search the Pubmed/Medline international database (**Supplementary Table 1**). Studies assessing professionals’ or patients’ preferences for RCC attributes, published from March 15, 2009 up to March 15, 2019, were reviewed. A total of eight studies with potential attributes for inclusion in the DCE were identified (**Supplementary Table 2**).

The final selection of characteristics for use in the DCE was based on a focus group with eight oncologists and on semi-structured telephone interviews with five aRCC patients. The main purpose was to validate the potential attributes identified in the literature, to identify relevant attributes not retrieved, and to assess the comprehensibility of the attributes and levels proposed. As a result, five attributes, with a maximum of three levels each, were selected (**Table 1**).

**Table 1:** Attributes and levels included in the DCE.

Attribute	Level
Survival gain	3 years (36 months)
	1.5 years (18 months)
	6 months
Risk of SAEs	5%
	15%
	30%
HRQoL	Improved
	Maintained
	Worse
Administration mode	Oral 1-2 times a day
	Intravenous monthly
	Intravenous every 15 days
Monthly cost	€3,000
	€5,000
	€8,000

SAEs, Serious Adverse Events; HRQoL, Health-related Quality of Life.

#### Experimental Design and Survey Instrument

A paper- or web-based survey was completed by patients and oncologists, respectively. The patient survey included sociodemographic data (provided by the patient) and clinical variables (collected by the physician from medical records), and the choice tasks, self-reported by the patient (**Supplementary Table 3**). The oncologist survey included sociodemographic variables and the same choice sets as for patients (**Supplementary Table 4**).

An experimental design was constructed, consisting of a series of choice tasks from combinations of the attribute levels (called scenario alternatives), following ISPOR recommendations (30). Fractional factorial design (orthogonal main-effect matrix) generated 18 scenarios, with a mix-and-match algorithm used to generate the choice sets. To avoid participant fatigue, the 18 scenarios were divided in two blocks of nine multiple-choice sets, distributed across two versions of questionnaires. The experimental task asked participants to choose their preferred treatment scenario. Additionally, as is generally performed in DCEs (40), an initial control scenario was included to check whether the participant understands the exercise. In this scenario, one treatment was clearly superior to the other, so participants who answered this question incorrectly were excluded from analysis (41). **Table 2** shows an example of the choice set.

**Table 2:** Example of a choice set presented to the study participants.

	Treatment A	Treatment B
**Treatment is administered…**	Intravenously every 15 days	Orally 1-2 times a day
**The probability of suffering serious adverse events (hospitalixzation, emergency, life-threatening…)**	5%	15%
**With the treatment, the quality of life (wellbeing, mobility, pain and fatigue control, social and family relations…)**	Is maintained	Worsens
**The survival gain with the treatment is…**	6 months	3 years
**The monthly treatment cost for the health system is…**	€3,000	€5,000
	I prefer treatment A	I prefer treatment B

### Analyses

Sociodemographic and clinical variables were described using absolute and relative frequencies of response for qualitative variables, and statistics of centrality and dispersion for quantitative variables.

Preferences were analyzed applying a mixed logit model [Stata software (42)], which accounts for preference heterogeneity among respondents (43). It yields both a mean effect and a standard deviation (SD) of effects across the sample. Attributes with quantitative levels (survival gain, probability of suffering serious adverse events [SAEs], and cost) were transformed into linear variables. In these cases, the utility value for the unit was obtained. The regression coefficients, referred to as part-worth utilities, are interpreted as the utilities associated with each level within an attribute. These coefficients are not directly comparable between attributes. To this end, the relative importance (RI) of an attribute over the range of attributes included in the experiment was estimated for each participant as the range of part-worth utilities of an attribute (difference in part-worth utilities between the best or preferred level and the worst or least preferred level of the same attribute), divided by the sum of all ranges across attributes and multiplying by 100 (44). The mean RI of each attribute was calculated for each group of participants (patients and oncologists). Furthermore, to assess differences and similarities between patients’ and oncologists’ preferences for aRCC treatment attributes, individual IRs between patients and oncologists were compared.

To identify possible explanatory variables in patients’ and oncologists’ preferences, subgroup analyses comparing individual RIs for each attribute were performed according to the following variables and cut-offs for patients: gender, median age, education level (primary studies vs. secondary education or higher), median time from diagnosis, number of comorbidities (≤ or > 1 comorbidity), number of treatments received for RCC (≤ or > 1 treatment), route (oral vs. others), and frequency of administration of current treatment (1/2 times a day vs. others); and for oncologists: gender, median age, median years of professional experience, and median years in RCC management.

For comparisons, Student’s t-test or the Mann-Whitney U test were used, according to data distribution. For all statistical tests, statistical significance was set at p<0.05.

The maximum acceptable risk (MAR) that participants were willing to trade-off for a treatment benefit was estimated as the quotient between the utility associated with a clinical benefit attribute (1 month survival gain) and the utility associated with a 1% increased risk of SAEs (45, 46).

Willingness to pay (WTP) for a given clinical benefit (increased survival or reduced likelihood of SAEs), was estimated as the ratio of the partial utility of the attribute levels and the partial utility per incremental cost (47).

## Results

### Sociodemographic Characteristics of Participants

A total of 105 aRCC patients (77.1% male, mean age of 65.9 years [SD: 10.4], mean duration from RCC diagnosis 6.3 years [SD: 6.1]) and 67 oncologists (52.2% male, mean age 41.9 years [SD: 8.4], mean length of professional experience 13.6 years [SD: 7.8] and of experience in RCC management 10.2 years [SD: 7.5]) were included in the final data analysis (**Table 3**).

**Table 3:** Sociodemographic characteristics of patients and oncologists.

Characteristics	Data
**Patients, n**	105
Age, years (n=104)	
Mean (SD)	65.9 (10.4)
Median (Q1-Q3)	65.2 (59.3-74.0)
Gender, %	
Female	22.9
Male	77.1
Visit to the health center, mean (SD)	
Distance (km), (n=104)	18.1 (23.0)
Time spent (min)	26.4 (16.9)
Transport cost (€), (n=103)	5.4 (6.6)
Other costs (€), (n=90)	5.1 (9.0)
Education level, %	
Primary education	44.8
Secondary education	34.3
Vocational training or other similar	3.8
University	16.2
MD, PhD	1.0
Employment situation, %	
Active	63.5
Non-active	22.9
Economic status (€/month), %	
<1000	28.6
1000-2000	39.0
>2000	20.0
No answer/don`t know	21.4
Time from RCC diagnosis, years	
Mean (SD)	6.3 (6.1)
Median (Q1-Q3)	4.3 (1.7-8.8)
Comorbidities	
Charlson index, mean (SD)	9.0 (1.7)
≤ 1 comorbidity (%)	50.5
Performance status (Karnofsky index, 0-100), %	
100 (normal)	43.8
90 (normal activity with signs of disease)	36.2
80 (normal activity with some effort)	17.1
70 (unable to work)	1.0
60 (requires assistance occasionally)	1.9
<50	0
Duration of current treatment, mean years (SD) (n=99)	1.5 (1.9)
Number of treatments received for RCC, %	
1	39.0%
≥1	61.0%
Route of administration of current treatment, % (n=99)	
Oral	73.7
Other	26.3
Frequency of administration of current treatment, % (n=99)	
1/2 times a day	67.7
Others	32.3
**Oncologists, n**	67
Age, years	
Mean (SD)	41.9 (8.4)
Median (Q1-Q3)	40.0 (35.0-49.0)
Gender, %	
Female	47.8
Male	52.2
Length of professional experience, years (n=66)	
Mean (SD)	13.6 (7.8)
Median (Q1-Q3)	11.5 (8.0-18.0)
Length of professional experience in RCC, years (n=66)	
Mean (SD)	10.2 (7.5)
Median (Q1-Q3)	9.0 (4.0-15.0)
Type of hospital, %	
<200 beds	7.5
200-500 beds	43.3
501-1000 beds	31.3
>1000 beds	17.9

Patients n=105 and oncologists=67, except when indicated.

### Preferences for RCC attributes

Items showing statistical significance (p<0.05) in both patients’ and oncologists’ preferences included levels of survival gain, SAE risk, level of intravenous administration every 15 days and worsening of HRQoL. Additionally, treatment cost was statistically significant (p<0.05) in the preferences of the oncologist group (**Table 4**). The signs of the coefficients indicate how this influence translates into preferences. In the case of SAEs, the results showed a greater preference for lower-risk scenarios. Survival gain coefficients indicated a greater preference for more years of survival. Intravenous administration every 15 days was less preferred than oral administration, and worsening of HRQoL was less preferred than improvement. The cost coefficients showed a greater preference for lower cost (only in the oncologist group). The main results of the mixed logit model are shown in **Table 4**.

**Table 4:** Utility scores in RCC patients and oncologists.

Attribute	Patients			Oncologists		
	Utility	Standard error	p-value	Utility	Standard error	p-value
Administration						
Oral 1-2 times/day^	0	–	–	0	–	–
			0.813			
IV monthly	-0.049	0.207		0.016	0.397	0.968
IV every 15 days	-0.788	0.233	0.001*	-1.605	0.520	0.002*
Serious treatment AEs						
Per unit	-0.047	0.009	<0.001*	-0.063	0.018	<0.001*
5%	-0.233	–	–	-0.315	–	–
15%	-0.699	–	–	-0.944	–	–
30%	-1.398	–	–	-1.887	–	–
HRQoL						
Improved^	0	–	–	0	–	–
Maintained	-0.587	0.226	0.009	-0.645	0.453	0.154
Worse	-3.367	0.528	<0.001*	-2.623	0.727	<0.001*
Survival gain						
Per unit	0.138	0.019	<0.001*	0.266	0.045	<0.001*
3 years	4.983	–	–	9.589	–	–
1.5 years	2.492	–	–	4.795	–	–
6 months	0.831	–	–	1.598	–	–
Monthly cost						
Per unit	0.000	0.000	0.779	-0.000	0.000	0.031*
€3,000	-0.032	–	–	-0.482	–	–
€5,000	-0.053	–	–	-0.804	–	–
€8,000	-0.085		–	-1.286	–	–

Part-worth utilities within attributes (utilities associated with each attribute level).

AEs, adverse events; IV, intravenous; *p < 0.05 statistically significant; ^Reference levels.

**Figure 1:**
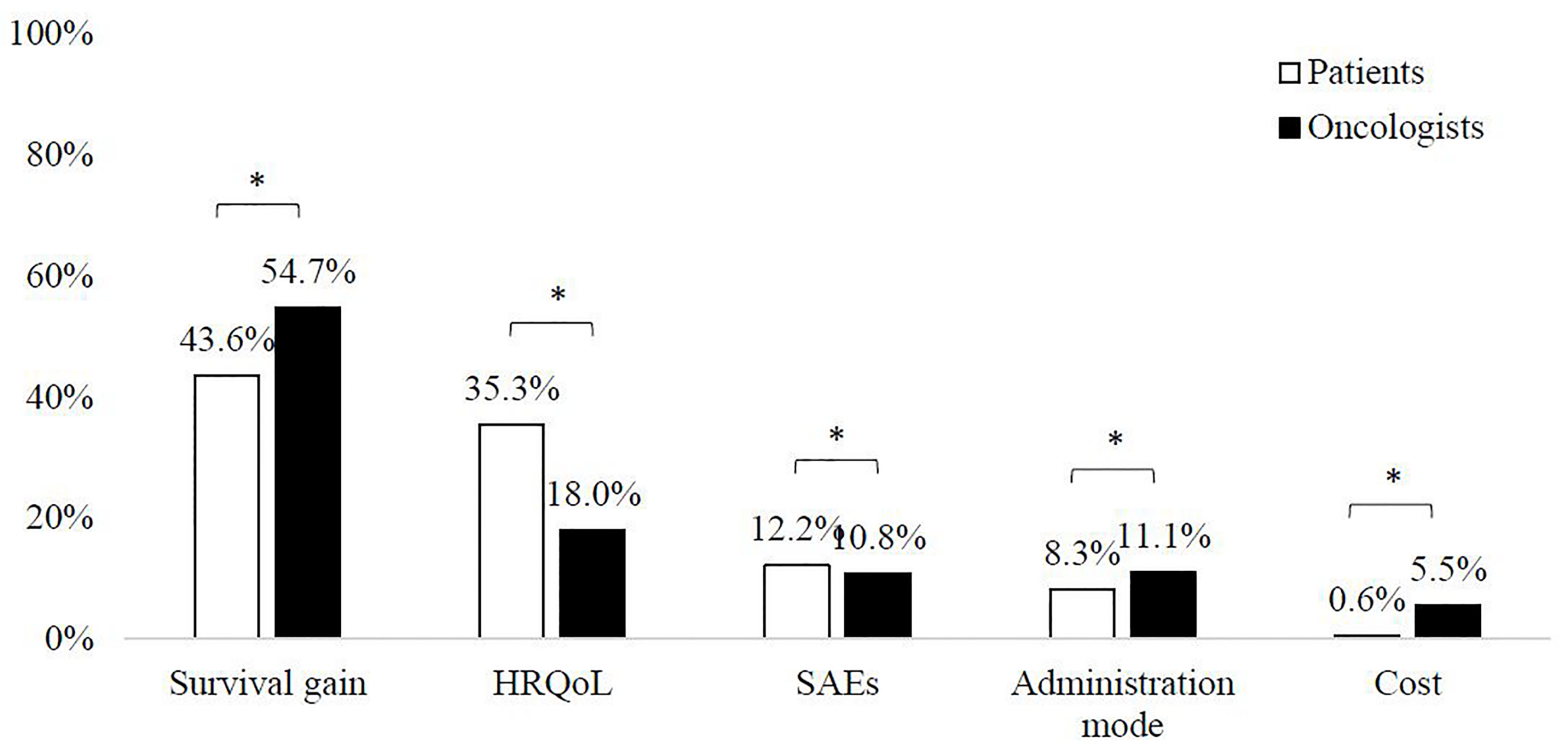
Relative importance of attributes *Significant difference between patients and oncologists (p < 0.05).

Among all the attributes studied, both patients and oncologists assigned the highest RI to survival gain (43.6% and 54.7% respectively), followed by HRQoL (35.3% and 18.0%, respectively). Cost was the attribute with the lowest RI for both groups (0.6% and 5.5%, respectively) (**Figure 1**). Significant differences were found in the RI assigned to all attributes between patients and oncologists (p<0.05) (**Figure 1**).

### Determinants of Preferences

Regarding subgroup analyses to identify possible explanatory variables in patients’ and oncologists’ preferences, it was found that patients with less time since diagnosis (< 4 vs. 4 years) and oncologists with less professional experience (< 11 vs. ≥ 11 years) assign higher RI to SAEs, while patients with a lower education level (primary studies vs. secondary education or higher) assign higher RI to survival and lower RI to administration mode and treatment cost (all p<0.05). No other differences were observed (**Supplementary Table 5**).

### Risk Tolerance and Willingness to Pay

Patients and oncologists were willing to accept an increase of 3.0% and 4.2% in SAE risk, respectively, in exchange for a one-month survival gain.

WTP was higher among patients than oncologists for increasing survival or lowering SAE risk (**Table 5**).

**Table 5:** Willingness to pay (WTP).

Attribute	Unit	Patients’ WTP	Oncologists’ WTP
**Survival gain**	↑1 month	€13,058.8	€1,656.5
**SAE risk**	↓1%	€4,395.7	€391.3

WTP for each attribute was estimated dividing the part-worth utility of the attribute levels (in this case, survival gain [per month] and SAE Risk [1%]) by the part-worth utility of the additional cost (per €); and expressed in absolute value.

## Discussion

The management of patients with aRCC has changed dramatically over the past few years as a result of the availability of multiple active agents. The near future will bring data on new agents and combinations of therapies administered to complement or even synergize with approved drugs and molecules currently under development. Hence, practitioners face new questions and challenges such as how to select the best drug for a given patient at a particular time. In this context, patients, physicians, and payers are seeking the highest value care for each patient, hence tools to compare the value of therapies have become necessary (1).

Stated preference surveys, such as DCE, are one of the most reliable and valid techniques available for quantifying participants’ preferences in healthcare (48). Accumulated evidence on patients’ and healthcare professionals’ preferences for RCC treatment characteristics is scarce. Greater knowledge of these preferences could help to identify relevant treatment characteristics for patients and healthcare professionals, improve patient management and encourage shared decision-making (49–51).

In this study, survival gain and improved HRQoL were the treatment attributes most highly valued by patients and oncologists. SAE risk was the third attribute in importance for patients, over administration mode. For oncologists, SAE risk and administration mode had similar RI. Cost of treatment (for the health system) was the attribute with lowest value for both groups. These results suggest that, in the RCC treatment decision, survival gain and improved HRQoL are essential for both patients and oncologists. Clinical benefit is an important part of value assessment, and in aRCC, overall survival has become the primary indicator of patient benefit. Beyond the general treatment goal of patients living longer, patient-specific factors also require consideration in the assessment of value (1). In this regard, HRQoL is increasingly recognized as a crucial aspect in overall patient outcomes (52). These factors have a major influence on decision-making and, hence, on the determination of the value of a treatment (1). Nonetheless, our results point out that improved HRQoL continues to be much more relevant for patients than for oncologists.

Previous studies have shown different results regarding patient preferences in RCC. Similar to our findings, Wong et al. and Mohamed et al. (31, 33) found that efficacy (PFS) was the most important attribute; while Park et al. (53) observed that patients considered AEs (hand-foot skin reaction, bone marrow suppression, gastrointestinal perforation, and bleeding) more important than PFS. Patients seemed to give greater importance to AEs that are more prevalent, while more serious but less prevalent AEs were considered less important (31, 33).

Preferences regarding treatment efficacy and AEs depend on both the magnitude of the benefit/risk and the patient’s own experience. Thus, patients who experience AEs of treatment tend to require greater clinical benefit than those who do not (54). In our study, patients with a longer time since diagnosis (and oncologists with longer professional experience) assigned lower importance to SAEs. This might implicate that these patients weigh the risks and benefits differently, in that they appear to accept more AEs as the price for being able to spend as much time as possible with their partners (24). In addition, it has been described that the weight of preferences may vary over the course of treatment (54). On the other hand, patients with a lower education level assigned greater importance to survival and a lower value to the administration mode and treatment cost. Other studies have also found an influence of educational level on preferences; for example cancer survivors’ preference for patient-centered care was associated with a higher educational level (55), the preference for the format in which prognostic information is provided for advanced cancer patients was influenced by the number of years of school education (56), and multiple sclerosis patients with higher education were less concerned about adverse events (57).

Notably, despite the key role of healthcare professionals in shared decision-making and treatment prescription in RCC, very few studies address the preferences of healthcare professionals. Park et al. found that the preferences of patients and healthcare professionals differed significantly. While patients gave more importance to AEs (as previously mentioned), physicians assigned a higher value to PFS, followed by route of administration and skin reactions. In fact, compared to patients, physicians would be willing to accept almost 10 times the risk of skin reaction and bone marrow suppression (1.3% vs. 9.6% and 1.0% vs. 11.8%, respectively) (53). In our study, the RI assigned to all treatment attributes was significantly different between the two groups of participants, with HRQoL and SAE risk being more highly valued by patients, and survival gain, administration mode and treatment cost more highly valued by oncologists. Therefore, given the lower value attributed to SAEs, oncologists were willing to accept a slightly higher increase in SAE risk than patients in exchange for 1-month survival gain (4.2% oncologists and 3.0% patients). In contrast, Gonzalez et al. showed that although efficacy was the most important attribute for both physicians and patients, the former gave greater importance to fatigue, skin reactions or other attributes such as co-payment (22). These results would be in line with those observed by Lawrence et al., in which the survival times judged sufficient to warrant treatment (e.g. adverse effects) were significantly higher among physicians (9) compared to patients (54). Of note, our study specifically addressed SAEs and not mild or general AEs, unlike these latter studies. Overall, these results highlight the relevance for physicians to recognize that their patients may have preferences that differ from their own when considering the potential benefits and harms of treatment.

In addition, given the high costs of these new treatments, physicians need to assess which drugs or combinations provide the best value for patients and the healthcare system and are thus worth pursuing (1). In publicly-funded National Healthcare Systems, prescribing physicians can be considered as serving a dual role, acting as patients’ advocates but also as society’s gatekeepers of the use of resources (58). We found that, despite cost (for the healthcare system) being the attribute with lower RI, it has an influence on oncologists’ (although not for patients) preferences. Consequently, WTP for survival gain or risk reduction was higher among patients.

The current study is subject to certain limitations. Although DCE is the recommended approach and is widely used to assess patient preferences for treatment characteristics, there is always a risk of a gap between stated and revealed preferences (30, 59); therefore, the study participants might make other choices in real life. Additionally, other attributes not included may also be relevant. However, this potential bias was prevented by selecting the most important attributes from the literature and using the input of an expert focus group (oncologists) and interviews with RCC patients. The selection of different levels may provide other utility values and relative importance. Another limitation is the inherent selection bias of getting the information from patients and oncologists willing to participate in the study, whose views might differ from those of other patients or oncologists. Finally, sample size requirements in DCE are also a controversial issue. No general recommendations exist, so sample sizes differ substantially between studies and the method used is not usually reported (48). The sample of participants in this study was representative of the number of Spanish RCC patients and Spanish oncologists experts in RCC. In this regard, the findings of the study significantly reflect preferences for RCC treatment attributes in Spain and should be interpreted within the context of the study. Above all, the comparison between patients’ subjective thoughts and oncologists’ data increases the strength of the results and provides new insights into the scarcely studied subject of oncologists’ preferences in RCC.

In summary, this study shows that, for both aRCC patients and oncologists, the most important treatment attributes are survival gain and improved HRQoL. Remarkably, SAE risk and administration mode are also important attributes to consider, while treatment cost is of least relevance. Patients would be more willing to accept that the health system should assume higher costs for improving survival and reducing SAE risk than oncologists. The results of the study can contribute to improved decision-making in the selection of an appropriate RCC treatment.

## Data Availability Statement

The original contributions presented in the study are included in the article/**Supplementary Material**, further inquiries can be directed to the corresponding author.

## Ethics Statement

The studies involving human participants were reviewed and approved by Ethics Committee of the Hospital General Universitario Gregorio Marañon (Madrid, Spain). Patients provided their written informed consent to participate in the study.

## Author Contributions

OF, ML-Q, GC, DSdP, ÁP, LB, AG, OE, and SB contributed equally to the design of the research and to data retrieval. CG-R and SA contributed equally to the conception and design of the research, the analysis and interpretation of the data and drafted the manuscript. VP and CP-S contributed equally to the conception and design of the research and to data interpretation. All authors critically reviewed the manuscript, agree to be fully accountable for ensuring the integrity and accuracy of the work, and read and approved the final draft.

## Funding

The study was funded by Bristol Myers Squibb.

## Conflict of Interest

OF has performed a consulting or advisory role for Astellas Pharma, Roche, Pfizer, Bristol-Myers-Squibb, Sanofi, EUSA Pharma, Sanoffi; has received speaking honoraria form Pierre-Fabre, Novartis, Bristol-Myers-Squibb, Ipsen, Roche, Astellas Pharma, Bayer, Janssen; and has received travel/accommodation expenses from Bristol-Myers-Squibb, Ipsen, Astellas. SB has received travel grants and/or honoraria for educational activities from BMS, Roche, Pfizer and MSD. ML-Q has participated in speaker’s bureaus for Roche, Boehringer Ingelheim, Janssen-Cilag, Ipsen, MSD, Novartis, Astra Zeneca, Lilly, Pfizer, Astellas Pharma; has performed a consulting or advisory role for MSD, AstraZeneca, BMS, Boehringer Ingelheim, Roche, Ipsen, EUSA Pharma, Eisai, Pfizer; and has received travel and accommodation expenses from MSD, BMS, AstraZeneca, Pfizer, Roche, Lilly. ÁP has received funding for research, travel grants and/or speaker fees from Pfizer, Novartis, EUSA Pharma, Ipsen, BMS, MSD, Merck, Janssen, Astellas, Bayer, Sanofi, Clovis and Roche. AG has received honoraria for educational activities from Bristol-Myers Squibb, Eusa Pharma, Ipsen, MSD, Pfizer and Roche-Genentech. CG-R and SA work for an independent research entity and have received fees for their contribution to the development of the project and the writing of the manuscript. VP and CP-S work at Bristol-Myers Squibb.

The remaining authors declare that the research was conducted in the absence of any commercial or financial relationships that could be construed as a potential conflict of interest.

The reviewer RMB declared a past co-authorship with the authors OF and ÁP to the handling editor.

## Publisher’s Note

All claims expressed in this article are solely those of the authors and do not necessarily represent those of their affiliated organizations, or those of the publisher, the editors and the reviewers. Any product that may be evaluated in this article, or claim that may be made by its manufacturer, is not guaranteed or endorsed by the publisher.
