# Supplementary material for: Preferences for Renal Cell Carcinoma Pharmacological Treatment: A Discrete Choice Experiment in Patients and Oncologists
Source: Front Oncol. 2022 Jan 7;11:773366. doi: 10.3389/fonc.2021.773366 (PMC8777125; doi:10.3389/fonc.2021.773366)
Supplement: Supplementary file 1 [file Table_1.docx]

Supplementary Material

Supplementary Table 1: Search terms and strategy in Medline/Pubmed

| Disease term | |
| --- | --- |
| #1 | "Renal Cell Carcinoma” [Mesh/All fields] |

| Treatment and decisión-making terms | | |
| --- | --- | --- |
| #2 | | "treatment” [All fields] |
| #3 | | "management" [All fields] |
| #4 | | "drug therapy" [All fields] |
| #5 | | "medication" [All fields] |
| #6 | | “patient centered decision making” [All fields] |
| #7 | | “shared decision making” [All fields] |
| #8 | | "health state" [All fields] |
| Preferences studies terms | | |
| #9 | "conjoint analysis” [All fields] | |
| #10 | "choice model" [All fields] | |
| #11 | "stated preference" [All fields] | |
| #12 | “DCE” [All fields] | |
| #13 | “discrete choice” [All fields] | |
| #14 | “decision analysis” [All fields] | |
| #15 | “preference” [All fields] | |
| #16 | “multi-criteria decision analysis” [All fields] | |
| #17 | “multi-attribute utility” [All fields] | |
| #18 | “analytic hierarchy process” [All fields] | |
| #19 | “trade-off” [All fields] | |
| #20 | “risk-benefit trade-off” [All fields] | |
| #21 | “preference weight” [All fields] | |
| #22 | “willingness to pay” [All fields] | |
| #23 | “WTP” [All fields] | |
| #24 | “willingness to accept” [All fields] | |
| #25 | “maximum acceptable risk” [All fields] | |
| Search strategy | | |
| #1 AND (#2 OR #3 OR #4 OR #5 OR #6 OR #7 OR #8) AND (#9 OR #10 OR #11 OR #12 OR #13 OR #14 OR #15 OR #16 OR #17 OR #18 OR #19 OR #20 OR #21 OR #22 OR #23 OR #24 OR #25) | | |

Supplementary Table 2: Publications included in the systematic literature review

| Publication |
| --- |
| Escudier B, Porta C, Bono P, Powles T, Eisen T, Sternberg CN, et al. Randomized, controlled, double-blind, cross-over trial assessing Treatment Preference for Pazopanib Versus Sunitinib in Patients With Metastatic Renal Cell Carcinoma : PISCES Study. J Clin Oncol. 2014;32(14). |
| Park M, Jo C, Bae EY, Lee E. A Comparison of Preferences of Targeted Therapy for Metastatic Renal Cell Carcinoma between the Patient Group and Health Care Professional Group in South Korea. Value Heal. 2012;15:933–9. |
| Wong MK, Mohamed AF, Hauber AB, Yang J, Liu Z, Rogerio J. Patients rank toxicity against progression free survival in second-line treatment of advanced renal cell carcinoma. J Med Econ. 2012;15:1–14. |
| Mohamed AF, Hauber Brett A, Neary MP. Patient Benefit-Risk Preferences for Targeted Agents in the Treatment of Renal Cell Carcinoma. Pharmacoeconomics. 2011;29(11):977–88. |
| Mansfield C, Srinivas S, Chen C, Hauber AB, Matczak E, Sandin R, et al. The effect of information on preferences for treatments of metastatic renal cell carcinoma. Curr Med Res Opin. 2016;32(11):1827–38. |
| González JM, Doan J, Gebben DJ, Boeri M, Fishman M. Comparing the Relative Importance of Attributes of Metastatic Renal Cell Carcinoma Treatments to Patients and Physicians in the United States: A Discrete-Choice Experiment. Pharmacoeconomics. 2018;36(8):973–86. |
| Blinman PL, Davis ID, Martin A, Troon S, Sengupta S, Hovey E, et al. Patients’ preferences for adjuvant sorafenib after resection of renal cell carcinoma in the SORCE trial: what makes it worthwhile? Ann Oncol. 2018 Feb;29(2):370–6. |
| Lawrence NJ, Martin A, Davis ID, Troon S, Sengupta S, Hovey E, et al. What Survival Benefits are Needed to Make Adjuvant Sorafenib Worthwhile After Resection of Intermediate- or High-Risk Renal Cell Carcinoma? Clinical Investigators’ Preferences in the SORCE Trial. Kidney Cancer. 2018 Aug;2(2):123–31. |

Supplementary Table 3: Patient Case report form

| **PATIENT'S SOCIODEMOGRAPHIC VARIABLES**  **Please mark with an X or write in the boxes, as appropriate:** | | | | | | | | | | |  |
| --- | --- | --- | --- | --- | --- | --- | --- | --- | --- | --- | --- |
| **DATE OF BIRTH:**   \|  \| \| --- \| \| \|  \|  \|  \|  \|  \|  \|  \|  \| \| --- \| --- \| --- \| --- \| --- \| --- \| --- \| --- \| \| \| (Day/month/year) \| | | | **SEX:**  Male  Female | | | **REGION**  _________________________________ | | | | |  |
| NIVEL EDUCATIVO:  Primary education (EGB or similar).  Secondary education (BUP, COU, ESO or similar)  University studies  Third-cycle studies (master's degree, PhD, etc.)  Others__________________________ | | | | | | | | | | |  |
| EMPLOYMENT STATUS (select one option):  Student  Domestic work  Unemployed  Employed full time  Part-time employee  Self-employed  Employed but on temporary sick leave due to renal cell carcinoma (RCC) since (day/month/year)________________  On permanent sick leave due to RCC since (day/month/year)____________________  Early retirement due to RCC since (day/month/year) ____________________  Early retirement due to other causes since (day/month/year) _____________________  Retired since (day/month/year)____________________ | | | | | | | | | | |  |
| ECONOMIC SITUATION (approximate monthly income):  Less than 1000 €/month  1000 - 2000 €/month  More than 2000 €/month  Don't know/no answer | | | | | | | | | | |  |
| TRAVEL TO HOSPITAL  Approximate distance: __________ Km  Approximate time: ____________ min  Means of transportation: On foot  Public transportation  Car  Other (specify)________  Approximate cost: ______________  Other costs derived from the visit to the hospital (such as food, parking...): ______________€. | | | | | | | | | | |  |
| PATIENT CLINICAL VARIABLES | | | | | | | | | | |  |
| DATE OF DIAGNOSIS of the patient's RCC by the SPECIALIST:  (if you do not know the day and/or month, mark the corresponding boxes with an X). | | | | | | | \|  \| \| --- \| \| \|  \|  \|  \|  \|  \|  \|  \|  \| \| --- \| --- \| --- \| --- \| --- \| --- \| --- \| --- \| \| \| (Day/month/year) \| | | | |  |
| CHARLSON INDEX   \| **Age** \|  \| \| --- \| --- \| \| Coronary artery disease \|  \| \| Congestive heart failure \|  \| \| Peripheral vascular disease \|  \| \| Cerebrovascular disease \|  \| \| Dementia \|  \| \| Chronic pulmonary disease \|  \| \| Connective tissue disease \|  \| \| Peptic ulcer disease \|  \| \| Mild Liver disease \|  \| \| Moderate to severe renal disease \|  \| \| Hemiplegia \|  \| \| Diabetes without end organ disease \|  \| \| Diabetes with end organ disease \|  \| \| Moderate to severe liver disease \|  \| \| Tumor \|  \| \| Leukemia \|  \| \| Lymphoma \|  \| \| Metastatic solid tumor \|  \| \| AIDS \|  \| | | | | | | | | | | |  |
| FUNCTIONAL STATUS (KARNOFSKY)   \| 100 \| Normal; no complaints; no evidence of disease. \| \| --- \| --- \| \| 90 \| Able to carry on normal activity; minor signs or symptoms of disease. \| \| 80 \| Normal activity with effort; some signs or symptoms of disease. \| \| 70 \| Cares for self; unable to carry on normal activity or to do active work. \| \| 60 \| Requires occasional assistance, but is able to care for most of their personal needs. \| \| 50 \| Requires considerable assistance and frequent medical care. \| \| 40 \| Disabled; requires special care and assistance. \| \| 30 \| Severely disabled; hospital admission is indicated although death not imminent. \| \| 20 \| Very sick; hospital admission necessary; active supportive treatment necessary. \| \| 10 \| Moribund; fatal processes progressing rapidly. \| \| 0 \| Dead \| | | | | | | | | | | |  |
| **RCC TREATMENT-RELATED VARIABLES** | | | | | | | | | | | |
| Please indicate the name of the ACTIVE INGREDIENT that the patient is CURRENTLY RECEIVING, the frequency, route of administration and the date on which the treatment started. (ANSWER ONLY IF THE PATIENT IS CURRENTLY RECEIVING TREATMENT) | | | | | | | | | | | |
| Active ingredient | Frequency of administration | | | | Route of administration | | | | Start Date | | |
|  |  | | | |  | | | | \|  \| \| --- \| \| \|  \|  \|  \|  \|  \|  \|  \|  \| \| --- \| --- \| --- \| --- \| --- \| --- \| --- \| --- \| \| \| (Day/month/year) \| | | |
| Please indicate the name of the ACTIVE INGREDIENTS that the patient has RECEIVED PRIOR TO CURRENT TREATMENT, including frequency, route of administration and start and end date. (ANSWER ONLY IN CASE THE PATIENT HAS RECEIVED PREVIOUS TREATMENT) | | | | | | | | | | | |
| Active ingredient | | Frequency of administration | | Route of administration | | | | Start Date (Day/month/year) | | End Date (Day/month/year) | |
|  | |  | |  | | | |  | |  | |
| If the patient has changed or prematurely terminated treatment for CRC, please indicate the PRIMARY REASON for ENDING TREATMENT IMMEDIATELY PRIOR to the current treatment (or last treatment, if not currently receiving treatment). Check ONE of the options:  Lack of efficacy  Occurrence of adverse effects  Change the route of administration  Other (specify):___________________________________________________________ | | | | | | | | | | | |

Supplementary Table 4: Oncologist case report form

| **SOCIODEMOGRAPHIC** **VARIABLES** | | |
| --- | --- | --- |
| **Birth date:**   \|  \|  \|  \|  \|  \|  \|  \|  \| \| --- \| --- \| --- \| --- \| --- \| --- \| --- \| --- \| \| day \| \| month \| \| year \| \| \| \| | **Sex:**  Male  Female | **REGION:**  _________________________________ |
| Years of experience in oncology: ____________________ | | |
| Years of experience in the management of RCC: ____________________ | | |
| Other cancers treated: ____________________________ | | |
| Number of RCC patients attended in approximately one month: _____________ | | |
| Type of hospital in which you practice:  Group 1 (Small county hospital or basic general hospital): Less than 200 Beds.  Group 2 (Area hospital, medium size): 200-500 Beds  Group 3 (Large Hospital): 501-1000 Beds  Group 4 (Large hospital complex): More than 1000 Beds | | |

**Supplementary Table 5:** Subgroup analysis of the preferences (relative importance, RI) of patients and oncologists for the characteristics of RCC treatments

| **PATIENTS** | | | | | | |
| --- | --- | --- | --- | --- | --- | --- |
| **Attributes** | **Age (years)** | | | | | |
|  | **RI**  <65 (N=52) | ≥65 (N=53) |  | p^µ^ | | |
| Survival gain | 39.7% | 46.1% |  | 0.170 | | |
| HRQoL | 35.0% | 32.1% |  | 0.485 | | |
| Risk of SAEs | 13.3% | 11.4% |  | 0.333 | | |
| Administration mode | 9.1% | 8.6% |  | 0.838 | | |
| Cost | 2.9% | 1.7% |  | 0.164 | | |
| **Attributes** | **Gender** | | | | | |
|  | **RI**  Male (N=81) | Female (N=24) |  | | | p^µ^ |
| Survival gain | 42.4% | 45.0% |  | 0.348 | | |
| HRQoL | 33.9% | 32.4% |  | 0.588 | | |
| Risk of SAEs | 12.8% | 11.0% |  | 0.230 | | |
| Administration mode | 8.8% | 8.9% |  | 0.903 | | |
| Cost | 2.2% | 2.7% |  | 0.071 | | |
| **Attributes** | **Education level** | | | | | |
|  | **RI**  Primary education (N=47) | Secondary education (or others) (N=58) |  | | p^µ^ | |
| Survival gain | **47.6%** | **39.2%** |  | **0.041*** | | |
| HRQoL | 32.1% | 34.7% |  | 0.443 | | |
| Risk of SAEs | 11.4% | 13.1% |  | 0,059 | | |
| Administration mode | **7.6%** | **9.8%** |  | **0.025*** | | |
| Cost | **1.3%** | **3.2%** |  | **0.011*** | | |
| **Attributes** | **Time since RCC diagnosis** | | | | | |
|  | **RI**  <4 years (N=52) | ≥4 years (N=53) |  | p^µ^ | | |
| Survival gain | 40.3% | 45.6% |  | 0.408 | | |
| HRQoL | 35.2% | 31.9% |  | 0.501 | | |
| Risk of SAEs | **13.3%** | **11.4%** |  | **0.041*** | | |
| Administration mode | 9.0% | 8.7% |  | 0.228 | | |
| Cost | 2.2% | 2.5% |  | 0.359 | | |
| **Attributes** | **Number of comorbidities** | | | | | |
|  | **RI**  1 (N=53) | >1 (N=52) |  | p^µ^ | | |
| Survival gain | 42.5% | 43.5% |  | 0.985 | | |
| HRQoL | 33.0% | 34.1% |  | 0.768 | | |
| Risk of SAEs | 12.9% | 11.8% |  | 0.658 | | |
| Administration mode | 8.9% | 8.7% |  | 0.913 | | |
| Cost | 2.7% | 1.9% |  | 0.696 | | |
| **Attributes** | **Number of treatments for RCC received** | | | | | |
|  | **RI**  1 (N=41) | >1 (N=64) |  | p^µ^ | | |
| Survival gain | 46.8% | 40.5% |  | 0.094 | | |
| HRQoL | 31.6% | 34.8% |  | 0.217 | | |
| Risk of SAEs | 11.6% | 12.8% |  | 0.170 | | |
| Administration mode | 8.4% | 9.1% |  | 0.419 | | |
| Cost | 1.5% | 2.8% |  | 0.170 | | |
| **Attributes** | **Treatment administration route** | | | | | |
|  | **RI**  Oral (N=73) | Other (N=26) |  | p^µ^ | | |
| Survival gain | 44.0% | 41.1% |  | 0.874 | | |
| HRQoL | 32.8% | 35.4% |  | 0.594 | | |
| Risk of SAEs | 12.3% | 12.9% |  | 0.943 | | |
| Administration mode | 8.7% | 8.5% |  | 0.775 | | |
| Cost | 2.1% | 2.0% |  | 0.750 | | |
| **Attributes** | **Treatment administration frequency** | | | | | |
|  | **RI**  1/2 times a day (N=41) | Other (N=64) |  | p^µ^ | | |
| Survival gain | 44.2% | 41.3% |  | 0.863 | | |
| HRQoL | 32.5% | 35.5% |  | 0.601 | | |
| Risk of SAEs | 12.3% | 13.0% |  | 0.952 | | |
| Administration mode | 8.8% | 8.3% |  | 0.510 | | |
| Cost | 2.2% | 1.8% |  | 0.840 | | |
| **ONCOLOGISTS** | | | | | | |
| **Attributes** | **Age (years)** | | | | | |
|  | **RI**  ≤40 (N=32) | >40 (N=35) |  | p^µ^ | | |
| Survival gain | 53.4% | 55.0% |  | 0.890 | | |
| HRQoL | 18.4% | 17.9% |  | 0.920 | | |
| Risk of SAEs | 11.6% | 10.4% |  | 0.069 | | |
| Administration mode | 11.1% | 11.1% |  | 0.380 | | |
| Cost | 5.5% | 5.6% |  | 0.633 | | |
| **Attributes** | **Gender** | | | | | |
|  | **RI**  Male (N=35) | Female (N=32) |  | p^µ^ | | |
| Survival gain | 54.7% | 53.6% |  | 0.506 | | |
| HRQoL | 17.6% | 18.7% |  | 0.280 | | |
| Risk of SAEs | 11.0% | 10.9% |  | 0.547 | | |
| Administration mode | 11.1% | 11.2% |  | 0.598 | | |
| Cost | 5.5% | 5.6% |  | 0.831 | | |
| **Attributes** | **Experience in oncology (years)** | | | | | |
|  | **RI**  ≤11 (N=34) | >11 (N=33) |  | p^µ^ | | |
| Survival gain | 52.4% | 56.1% |  | 0.103 | | |
| HRQoL | 19.2% | 17.0% |  | 0.117 | | |
| Risk of SAEs | **11.6%** | **10.3%** |  | **0.033*** | | |
| Administration mode | 11.3% | 11.0% |  | 0.322 | | |
| Cost | 5.6% | 5.5% |  | 0.373 | | |
| **Attributes** | **Experience in RCC (years)** | | | | | |
|  | **RI**  ≤9 (N=32) | >9 (N=35) |  | p^µ^ | | |
| Survival gain | 53.5% | 55.0% |  | 0.633 | | |
| HRQoL | 18.5% | 17.7% |  | 0.782 | | |
| Risk of SAEs | 11.3% | 10.6% |  | 0.253 | | |
| Administration mode | 11.2% | 11.1% |  | 0.539 | | |
| Cost | 5.5% | 5.6% |  | 0.841 | | |

Cut-off points of the subgroups for age, time since RCC diagnosis, and time of professional experience and RCC experience have been stablished according to the median value of the variables.

^µ^Mann–Whitney U test; *p<0.05, statistically significant; HRQoL: health-related quality of life; RI: Relative Importance; SAEs: serious adverse events.
